# A Critical Study on the Impact of Dementia on Older People Undergoing Treatment in Care Homes

**DOI:** 10.7759/cureus.30056

**Published:** 2022-10-08

**Authors:** Hitaishi Aggarwal, Sarika Chaware, Hardik Aggarwal

**Affiliations:** 1 Psychiatry, Jawaharlal Nehru Medical College, Datta Meghe Institute of Medical Sciences, Wardha, IND; 2 Community Medicine, Jawaharlal Nehru Medical College, Datta Meghe Institute of Medical Sciences, Wardha, IND; 3 Medicine, All India Institute of Medical Sciences, Rishikesh, IND

**Keywords:** healthcare, dementia care homes, quality of life of dementia patients, impact of dementia on older people, dementia

## Abstract

Maximum healthcare needs for older people are complex due to diseases, comorbidities, or disabilities, including long-term or mental and physical health issues. Various residential and nursing care homes help care for older people, especially those with special medical needs. Among these special medical needs, dementia is one medical condition requiring exceptional care for the affected to prevent adverse effects of the situation they usually encounter. Dementia is a health condition that involves impairments to memory and thinking due to any injury or disease-causing damage to the brain. Older people suffer from different diseases, which cause cognitive disability and long-term ailments and directly affect patients' quality of life. Given the cognitive impairment dementia causes to older people, it is difficult for the care providers to accurately assess the impact on every individual to formulate a person-centered care plan. During the COVID-19 pandemic, due to administrative restrictions on social distancing to prevent transmission of this disease, caregivers and elderly persons feel tremendous mental stress, further aggravating their problems because of loneliness. Thus, there is a requirement to do the study and analyze the effects on older people to provide quality and person-centered care. Due to the above factors as significant challenges in the current context, there is an ardent need for the results of variegated studies besides a thorough analysis of available literature analyzed to provide proper evidence to the care providers. It will pave the way for understanding the actual impact of the condition in its natural context. In this regard, a literature review and the results of the studies are discussed. This research brings into the limelight all those factors in the context of previous studies and data analysis of the current situation.

## Introduction and background

Dementia is a health condition that involves impairments to memory and thinking due to any injury or disease-causing damage to the brain. Common challenges faced by dementia patients include memory loss (general), a sudden shift in behavior or mood change, confusion, loss of speaking ability, and trouble walking and in balance. These behavioral changes will lead to the social exclusion of the affected, resulting in agitation, anxiety, aggression, and depression in dementia patients [1].

The leading cause behind the disease is abnormal brain changes or brain cell damage, which negatively impacts behavior, memory, and thinking ability. Though this situation does not fall within the life cycle of aging, it has gained awareness globally due to its increased prevalence in recent times. Other risk factors for developing dementia include genetics and factors contributing to cardiovascular risk [2]. Further, the disability caused in the late stages of the condition is more critical. These kinds of problems need the provision of patients with person-centered treatment. In this situation, general healthcare services might not be adequate, and the caregivers might need the appropriate training to deliver effective care [3]. 

The maximum number of older people have agonized from cognitive disability, and a long-term disease caused by this condition considerably adversely affects the patient's regular cycle of living. Therefore, there is a need to do this particular study and evaluate the impact of the situation, especially on aged people, to provide quality and person-centered care [4]. One interdisciplinary approach is palliative care, practiced and meant to improve the quality of life, particularly for dementia patients. A lot is required from caregivers of the patients, and the duties can also be extensive. Education and awareness are the most crucial aspect for caregivers. Cognitive simulation therapy and reminiscence therapy are effective therapies to improve the quality of life and better management of symptoms in people with dementia [5]. 

**Figure 1:**
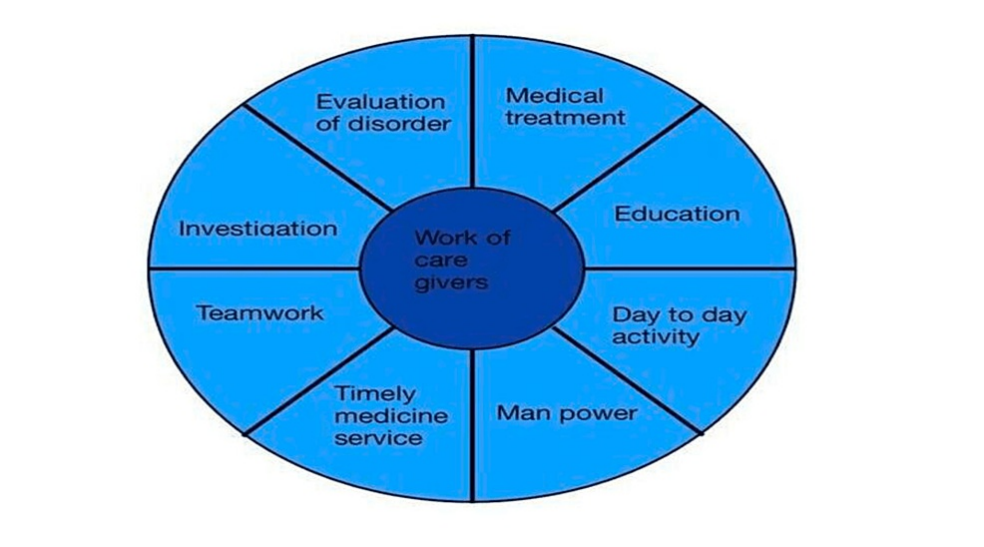
Work of caregivers [8]

Loneliness is a significant problem for most dementia patients, and this may trigger anxiety and depression. Hence it is critical to attend to the psychosocial needs of the patients to ensure they are emotionally more stable [6]. Further, it is crucial for healthcare professionals to recognize the different kinds of care required per the progression of the disease and its negative impact on the patients. Hence, a literature review is carried out to study the effect of the condition on older people [7]. The key objective of this research study is to discover the impact of dementia on the quality of life of older patients and compare the same as the disease progresses. The work of these caregivers is shown in Figure 1 below.

During the COVID-19 pandemic, caregivers felt tremendous stress in giving specialized care to elderly dementia patients due to maintaining social isolation to prevent the disease's spread and keep themselves lonely. Social activities and coordinated care for elderly patients were also affected. Various self-assessment methods were under trial to assess the mental status of caregivers as well as dementia elderly persons during the pandemic. From multiple databases, it was observed that the quality of life of caregivers was impacted severely due to the mental stress of loneliness during COVID-19. Further, studies in this regard will prepare us for a better way to deal with such situations in the future. The medical and psychological management of caregivers and dementia elderly patients can be improved. Telehealth may be a promising safe way to look after this vulnerable group of patients in the future [9].

In the pandemic, due to social isolation, elderly cognitive adults feel more anxious, angry, stressed, and withdrawn, which makes them susceptible to suicidal tendencies. Further, various stress triggers like frustration, boredom, insufficient information about the pandemic, and irregularity in food and medicine supply also affected their mental state adversely. Australian psychological society took the initiative to provide accurate information about social isolation needs, disease spread, hand hygiene, etc., to help elderly adults. Using modern technology like telehealth, the use of telephones for teaching and treating vulnerable elderly adults helps reduce the risk of suicide in them. Further, they advised that all elderly should have access to their families and medical services [10].

Elderly dementia people suffered severely during the COVID-19 pandemic and were more prone to depression, suicidal tendencies, anxiety, substance abuse, and post-traumatic stress disorders. Activities like book clubs, movies, therapy animals, and contact with family members using plastic protective barriers help them recover better post-pandemic era and relieve them from stressful conditions. Because of the cognitive state of elderly dementia, adults felt it difficult to adhere to the guidance of social distancing, hand hygiene, face masks, etc. Regular home visits and consultations with the help of psychiatrists or clinicians videoconferencing after hospital discharge also helped to cure this post-traumatic stress disorder. More stress was on improving existing health infrastructure to deal with the negative effects of mental health issues due to loneliness [11].

## Review

Epidemiology

It is crucial to know that dementia has a significant impact on the individual and the relationships, particularly older people, and leads to social isolation in these patients [12]. It is ranked the 9th most burdensome for people aged above 60 years. Furthermore, approximately 50 million people worldwide suffer from this neurological condition, and the figures are expected to rise three-fold by 2050 [13]. Health condition represents a significant reason for dependency among older adults. It is estimated that the societal cost of dementia is around 800 billion US dollars, i.e., more than 1% of the global gross domestic product [14]. It is estimated that globally about two people in every 100 of age above 65 years are suffering from the disease. Numbers are increasing to one-fifth of the people aged above 85 years. As per data, Finland has the highest disease prevalence, followed by the United Kingdom. Though the condition is mainly prevalent in older people, rarely is it not linked to aging. The disease has a low prevalence in people below 40 years of age and is considered rare in the people of 40 years-60 years age group. Currently, the global caseload is more than 55 million patients, and the cases are increasing at a rate of 10 million new patients diagnosed with the condition annually [15].

Methods

**Figure 2:**
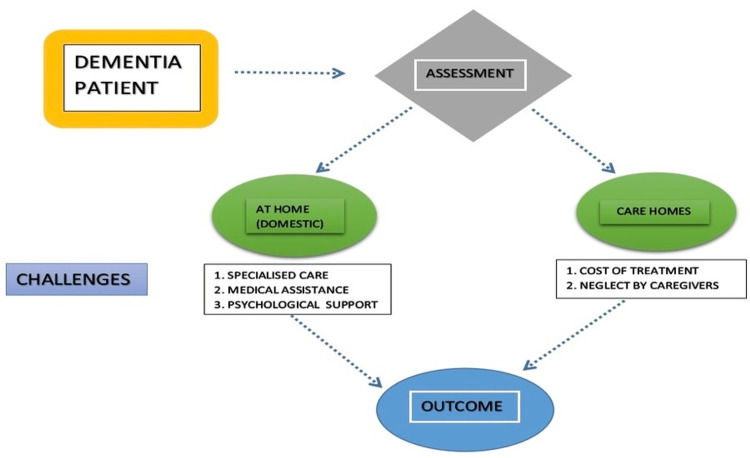
Challenges for outcome in managing dementia patient in elderly in both care homes and at home (domestic)

A researcher must comprehend how the study issue will be resolved to narrow down the number of relevant papers that can address it. This current study suggests gathering information on how dementia affects older individuals generally in terms of their standard of living [16]. It will assist medical practitioners in comprehending and addressing the crucial component of the research issue, namely the influence of a health problem on older people's general well-being. This would then enable caretakers to change their care plan following the demands of the residents [17]. In general, qualitative and quantitative approaches are used to acquire the information necessary for the study. This study used a qualitative approach and gathered information about the participants' perceptions and emotions which are chosen for data gathering because the research seeks to understand how dementia affects older persons [18]. To study the literature review on dementia patients, several databases are available. Cumulative index to nursing and allied health literature plus, PubMed, and EMBASE (Excerpta Medica Database) is used for the selected databases. Elderly individuals' in-home care, the effect of dementia, and patients' living conditions are among the terms included in the selection method [19]. Dementia care coordinators, with their skilled initial assessment at home and living situation, can easily guide caregivers with managing dementia elderly persons at home. Further, safe medical care and management, nutritional care, etc., can help them live with the highest quality of life for a long time. But in contrast, when family members do not understand the disease situation and its progression and are unable to provide their loved ones the desired care at home, rehabilitation facilities, nursing homes, and living homes with health worker assistance are the alternatives we use must consider [20]. Figure 2 gives a brief description of the outcome of managing dementia patients in the elderly in both situations.

Literature review

No cure has been developed for the condition except for reversible types. Though medical practitioners use some inhibitors in the disorder's early stages, the effect is marginal. According to research, psychological and creative treatments help treat agitation and violence in addition to all kinds of medications [21]. In the absence of effective new treatments, psychosocial or behavioral therapies are promoted to lessen the consequences of dementia due to the rising incidence of dementia, primarily in older individuals [22]. Given the cognitive impairment, dementia causes to older people, it is difficult for the care providers to accurately assess the impact on every individual to formulate a person-centered care plan. Further, the care of elderly adults with dementia also includes a variety of treatments, such as innovative therapy and pet therapy [23]. Therefore, it is crucial for healthcare professionals to establish proper treatments in the care plan by having a thorough grasp of how dementia affects individuals in both themes [24]. 

As mentioned earlier, assessing, evaluating, and contrasting the study findings with existing literature is necessary to give healthcare professionals the information required to comprehend the disease's actual effects and adjust their treatment plans accordingly. The first component of the data analysis will explain how the disorder generally affects older people's standard of living. The second stage will explain how this ailment affects the inhabitants of care homes and how they compare to the residence dwellers that need to be managed. The final level of data analysis will also connect with the solution to the research topic. Three articles were shortlisted from the database search to analyze the findings and understand the impact of dementia on older people. In the first investigation, the quality of life of older adults with dementia is evaluated by a comprehensive study (systematic review). The studies on the quality of life of dementia-affected older people published between 1995 and 2020 were systematically reviewed for this study. This study has examined 19 studies. The main goal of this study is to evaluate the quality of life, particularly in older adults with dementia. Hence, the studies reviewed have participants of age above 65 years [25].

This systematic review aims to evaluate the quality of life of elderly individuals with dementia and determine the variables that affect the quality-of-life results. The researchers within those studies employed five different quality-of-life instruments to collect respondent ratings. The quality of life in the Alzheimer's disease scale, which evaluated social, psychosocial, and behavioral functioning, was the most frequently utilized tool in the investigations; after that, the review recommended assessing the individuals' quality of life using self-esteem and proxy-rated levels. However, the study pointed out that there may be a bias in identifying several detrimental characteristics, such as sadness and restlessness, recorded in self-assessment questionnaires [26]. Although better quality of life is generally linked to a patient's improved intellectual performance, the review indicated that for individuals with dementia, higher quality of life values are linked to lower levels of knowledge and awareness of their condition so, in these situations, the high results are independent of any cognitive decline [27]. 

The main characteristics that negatively impact quality-of-life ratings are carers' higher infection burden and anxiety. Further, the individuals' quality of life levels were more significant due to prompt and appropriate pain control. The quality of life of people with dementia was severely impacted by recurrent admissions and other problems, including falls. Still, the above factors, highlighted by the study, are irrespective of the person seeking care from care homes or care provided by family members. According to the study of the current theme, people living with dementia's quality of life is influenced by pain treatment, patient guidance about not paying increasing emphasis to the condition's course, and the disease's devastating impact on the carers [28]. 

A review was conducted on another study focused on living with dementia in nursing homes. In the face-to-face survey, 12 respondents residing in three separate care facilities were subjected to unscripted interviews and field inspections every three months apart. Six participants lived in special dementia care facilities, whereas the study subjects did so in standard units. These interviews were structured around three key themes: connections with other inhabitants of the treatment facility and the employees, flourishing (a sensation of belonging), and purpose of making (offered in care facilities). The investigators identified four critical contributions from the patients' views on nursing facilities from the interview. The study made us understand that these dementia patients' could no longer reside at home, and the majority expressed satisfaction with their choice to live in healthcare institutions because most participants have stress due to separation from family, a former life, and a home. Furthermore, few participants have indeed mentioned the feeling that they have lost their sense of identity also. The participants admitted that they require assistance with most daily tasks; as a result, the staff and activities in healthcare facilities improve the residents' health. The interviews revealed that the individuals' lives depend most on the contacts from their close relatives [29]. 

This study found that a lack of understanding of how dementia affects patients' cognitive skills results in unprofessional behavior and the physical presence of personnel. It also became apparent how they interpreted what was happening in their immediate environment [30]. According to the study's observations, it is clear that older adults with dementia feel like they are losing their past in assisted living facilities and are entirely dependent on care personnel for their daily tasks [31]. Another study got reviewed to compare, focusing on the impact of dementia on older adults in residential homes and home-dwelling patients. It focuses on the quality-of-life difference between care home-dwelling and home dementia patients. This cross-sectional study was carried out on 78 residential homes and 115 home-dwelling participants, where trained and experienced nurses scored the quality of life and other related data on these dwelling participants. Various parameters were measured, mainly focused on patients' sleeping patterns, activity levels, and light exposure [32]. The findings were as follows: after categorizing the data according to disease activity, home-based participants displayed higher patients quality of life [33]. The elderly dementia patients who live at home have increased numbers of physical and social engagement and are less dependent on assistive devices and psychiatric drugs [34]. However, for the advantages and living conditions for older individuals, the study concluded that for older patients with mild dementia, remaining at home is preferable to keeping them in resident facilities [35].

A significant advantage among Indian dementia patients for improving their quality of life is that the elderly usually live with their families. Their families typically take care of them in this dementia situation in an ordinary family situation. The challenging problem for caregivers in joint family situations due to progressive memory impairment is forgetting recent conversations, recent events, telephone numbers, and names of close relatives. It results in depressive conditions, so they need supervised care for these daily basic needs. Even the family members caring for dementia patients must need psychological help and guidance to deal with such situations as family therapy, group therapy, and counseling. They feel tremendous stress while treating such elderly family members. They should know to treat problems like hypothyroidism and vitamin deficiency and monitor blood pressure. Treatment like anticoagulant therapy and medication like donepezil, rivastigmine, and galantamine are also beneficial in the early stages of this disease. All these measures help in improving the quality of life under supervision. Various strategies like improving lifestyle, proper diet, and activities enhancing mental abilities like reading, solving puzzles, sudoku, etc. help deal with these situations. Moreover, encouraging physical activities like yoga exercises and fixes like grab bars in the bathroom, carpets to prevent falls, etc., can help prevent the development of this dementia situation and further slow its progress in older people in joint family situations [36].

The dementia India report provides seven core strategies focusing on providing services for people diagnosed with dementia, creating awareness amongst people, and building healthcare teams for management of dementia for long-term care through community-based programs and training services to control dementia better [37]. For senior health problems, the Ministry of Health and Family Welfare of India has launched a National Programme for Health Care of the Elderly (NPHCE) during the 11th five-year plan, which deals with various health problems related to old age. This program aims to provide separate and specialized health to the elderly population. Another initiative of this program is to create awareness in sub-center and primary health centers on dementia. There is also a visit of healthcare workers for the care of bedridden older people and providing facilities to them and training to their families for their care. Combining primary and mental healthcare has successfully treated dementia cases in India [38]. Various aspects to be taken care of amongst caregivers in care homes are whether they are trained in first aid and cardiopulmonary resuscitation under challenging situations, working experience, training for dementia care, their reliabilities, availability, and replacement when sick [39].

## Conclusions

In this study, the linkage between factors considerably impacting dementia patients living in care homes and those staying with their families is observed. In care homes, healthcare professionals working as caregivers feel overburdened due to the rapid increase in dementia patient's load which results in poor management of such older patients resulting in depression and irritability. In contrast, elderly dementia patients staying with their families have a better outcome, fewer adverse effects, and a good standard of living. Regardless of disease progression, therapies adopted, and patient's waiting environment, dementia significantly impacts older people's lives due to the cognitive impairment caused by the disease. As many cures like creative and robotic pet therapies are being used to relieve symptoms and reduce disease progression, more research and analysis to find ways to improve the quality of life (free of permanent impairment) in dementia patients in care homes and residential homes is required. Hence research should focus on the methods of care therapies to ease symptoms, increase the sense of inclusion in society and avoid serious complications such as depression in dementia patients.
